# Evaluation of microarray data normalization procedures using spike-in experiments

**DOI:** 10.1186/1471-2105-7-300

**Published:** 2006-06-14

**Authors:** Patrik Rydén, Henrik Andersson, Mattias Landfors, Linda Näslund, Blanka Hartmanová, Laila Noppa, Anders Sjöstedt

**Affiliations:** 1 Department of Clinical Microbiology Division of Clinical Bacteriology, Umeå University, SE-90187 Umeå, Sweden; 2 Department of Mathematics and Mathematical Statistics, Umeå University, SE-90187 Umeå, Sweden; 3 Proteome Center for the Study of Intracellular Parasitism of Bacteria, Faculty of Military Health Science, University of Defence, Trebesská 1575, 50001 Hradec Králové, Czech Republic; 4 Department of Medical Countermeasures, Division of NBC-Defence, Swedish Defence Research Agency, SE-90182 Umeå, Sweden

## Abstract

**Background:**

Recently, a large number of methods for the analysis of microarray data have been proposed but there are few comparisons of their relative performances. By using so-called spike-in experiments, it is possible to characterize the analyzed data and thereby enable comparisons of different analysis methods.

**Results:**

A spike-in experiment using eight in-house produced arrays was used to evaluate established and novel methods for filtration, background adjustment, scanning, channel adjustment, and censoring. The S-plus package EDMA, a stand-alone tool providing characterization of analyzed cDNA-microarray data obtained from spike-in experiments, was developed and used to evaluate 252 normalization methods. For all analyses, the sensitivities at low false positive rates were observed together with estimates of the overall bias and the standard deviation. In general, there was a trade-off between the ability of the analyses to identify differentially expressed genes (*i.e*. the analyses' sensitivities) and their ability to provide unbiased estimators of the desired ratios. Virtually all analysis underestimated the magnitude of the regulations; often less than 50% of the true regulations were observed. Moreover, the bias depended on the underlying mRNA-concentration; low concentration resulted in high bias. Many of the analyses had relatively low sensitivities, but analyses that used either the constrained model (*i.e*. a procedure that combines data from several scans) or partial filtration (a novel method for treating data from so-called not-found spots) had with few exceptions high sensitivities. These methods gave considerable higher sensitivities than some commonly used analysis methods.

**Conclusion:**

The use of spike-in experiments is a powerful approach for evaluating microarray preprocessing procedures. Analyzed data are characterized by properties of the observed log-ratios and the analysis' ability to detect differentially expressed genes. If bias is not a major problem; we recommend the use of either the CM-procedure or partial filtration.

## Background

A large number of methods for low-level analysis of microarray data have been developed, but the relative merits of these methods are generally not easy to assess [1]. Analytical methods are commonly motivated by theoretical properties or how well they perform on simulated microarray data [2-4]. Neither approach is fully satisfactory, since they rely on model assumptions that are not necessarily supported by empirical studies. For real data, the true values are not known and therefore cannot be characterized and used for evaluation. Data from spike-in experiments, where the mRNA-ratios of a set of artificial clones are known, can be used to determine the relative merits of a set of analysis methods [1,5]. The design of a spike-in experiment needs to be based on assumptions of how real microarray data behave. However, these assumptions are generally less restrictive than the ones needed for simulating microarray data. The presented evaluation study used eight in-house produced spike-in microarrays (*The Lucidea array*) with approximately 10,000 spots, 4,000 of which were spiked at different concentrations, *i.e*., differentially expressed (DE) genes. In comparison with the spike-in study performed by Qin et al. [5], our spike-in array encompasses more DE-genes, allowing us to obtain reliable estimates of the methods' abilities to detect DE-genes (*i.e*. the methods' *sensitivities*).

Microarray studies are often used to screen for DE-genes. In this case, the sensitivity and specificity of the study are of interest. The *Receiver Operating Characteristic curve *(ROC-curve) shows the relationship between sensitivity and specificity and can be used to characterize the classification properties of a study [6]. Alternatively, pre-processed microarray data are sometimes used in so-called high-level analyses (*e.g*. cluster analysis and classification). In this case, the sensitivity and specificity of detection is no longer the most appropriate framework for evaluation. Rather, the properties of the normalized log-ratios need to be understood.

In this article, 252 normalization procedures were evaluated. We simultaneously evaluated three filtration methods, two techniques for background adjustment, seven scanning procedures, two ways of dealing with negative intensities, and four censoring approaches. The majority of these methods are well established, but we also introduced some novel ones: *partial filtration *handles data from spots not properly identified by the image analysis software, *C-spot inclusion *handles negative background adjusted intensities, and *censoring *moderate extreme ratios caused by weakly expressed genes.

## Results

### The general model

**Figure 1:**
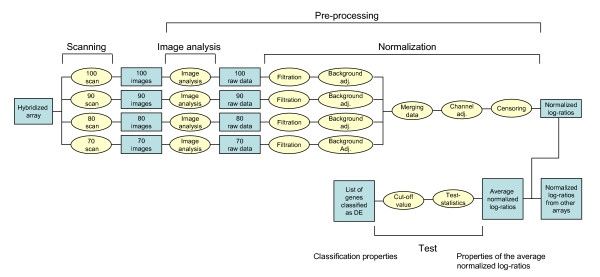
**Description of the evaluation study**. Theevaluation study was based on raw data from eight Lucidea arrays (Figure 2). All arrays were scanned at four settings and image analysis was applied to extract raw data. The data were normalized in five steps. Filtration and background adjustment were applied to each of the raw data sets. Three procedures for filtration and two procedures for background adjustment were considered. In the third step, data from the four scans were "merged" together. Seven merging procedures were considered; four of these were single scan procedures that used only data from a single scan. Two procedures for channel adjustment and four censoring approaches were considered. In total, 252 unique analyses were conducted. For each analysis the normalized log-ratios were used to conduct a test that generated a list of genes classified as DE. Properties of the average normalized log-ratios and the analyses' classification properties were used to characterize and evaluate the analyses.

We consider a two-channel cDNA-microarray experiment with a reference and treated channel. Let *μ *denote the true gene expression level of a gene. The raw intensities cannot be directly used to identify DE-genes, since they are influenced by systematic variation. Normalization aims to remove systematic variation and create *normalized log-ratios *that are used to calculate *test-statistics *that rank the genes according to how likely they are to be DE. Genes with test-statistics above a user defined cut-off value are classified as DE. For each gene and array the normalized log-ratio should be an observation of the true log-ratio of interest, *i.e*. log_2 _(*μ*_Treated _/*μ*_Reference _). The methods used for transforming raw data to normalized log-ratios constitute a *normalization procedure*. We consider raw data generated from arrays that have been scanned at four laser settings, where the normalization procedures involve filtration, background adjustment, merging data from different scans, channel normalization, and censoring (Figure 1).

### Design of spike-in microarray arrays

**Figure 2:**
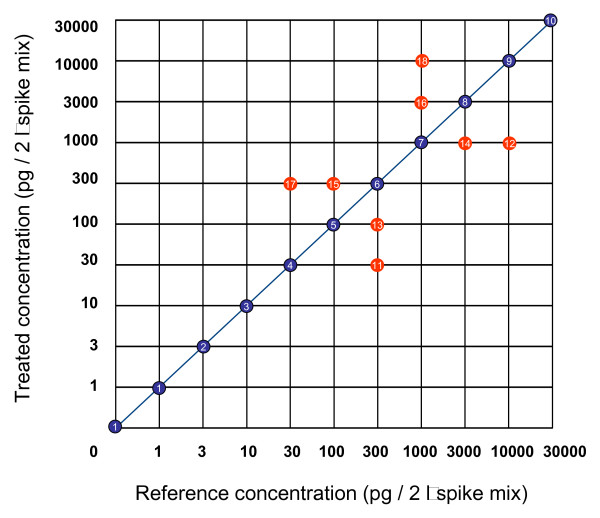
**Description of the Lucidea array**. The Lucidea array has 9,600 spots from 20 genes; each gene was replicated 480 times. Eight genes were spiked with different mRNA-concentrations in the treated and reference channels (*i.e*. the DE-genes, red dots 11–18). The remaining 12 genes were spiked equally in both channels at different mRNA-concentrations (*i.e*. the NDE-genes, blue dots 1–10). Three NDE-genes, two expressed at concentration zero and one at concentration one, were clustered together and are in this paper treated as one gene.

In a spike-in experiment, *control clones *(*i.e*. artificial clones designed to avoid cross-hybridization) are printed on an array. We will refer to control clones as genes even though they do not correspond to actual genes. Prior to labeling, the biological samples are spiked with control genes of known concentration. All other experimental steps are identical to a standard two-channel microarray experiment. The evaluation study presented in this paper used an in-house produced cDNA-array (*the Lucidea array*), consisting of 12 non-differentially expressed (NDE) genes and 8 DE-genes from the Lucidea Universal ScoreCard (Amersham Biosciences), where each gene was spotted 480 times. The NDE-genes were spiked with different RNA-concentrations ranging from zero to very high concentrations. The DE-genes were either three-fold or ten-fold up- or down-regulated, and were spiked with low or high RNA-concentrations; see Figure 2. Technical details about array production and experimental protocols are described in the methods section.

### Methods and tools for evaluation of spike-in microarray data

An analysis involving image analysis, normalization, and a test generates normalized log-ratios and test-statistics (*e.g*. the B-statistic [2] or the absolute value of the t-statistic). A gene is classified as DE if its test-statistic is above a user determined cut-off value *c*. The experiment's *sensitivity *is the proportion of DE-genes that are correctly classified. The *false positive rate *(FPR) is the proportion of NDE-genes that are falsely classified, while the *false discovery rate *(FDR) is the proportion of NDE-genes among the genes classified as DE.

The analyzed microarray data are characterized by the properties of the normalized log-ratios and their classification properties (Figure 1). The former is characterized by the ROC-curve, describing the relationship between the sensitivity and the FPR. If the purpose is to screen for DE-genes it is sufficient to consider the classification properties. However, unbiased estimates of the normalized log-ratios are important in order to understand the biology and are desirable when combining results from different techniques (*e.g*. cDNA-arrays, short-oligo arrays, and qRT-PCR). Furthermore, if the bias depends on the genes' mRNA-concentrations (*e.g*. the bias is higher for DE-genes expressed at low concentrations rather than at high concentrations), then results from high-level analyses such as clustering or classification can be misleading. We propose that the following properties should be considered when evaluating analysis methods:

I. High sensitivity at the user acceptable FDR.

II. The expected values of the average normalized log-ratios of DE-genes should be close to the true log-ratios, and the bias should not depend on the genes' mRNA- concentrations.

The relative importance of these properties depends on the purpose of the experiment.

The properties of the normalized log-ratios can be summarized with estimates of the overall bias and standard deviation. Consider an experiment with *r *DE-genes, where the *k*th gene is replicated *n*_*k *_times and has the true log-ratio. The *reflected bias *and the *selected bias *are measures of property II. The reflected bias is estimated by

b^DE=∑k=1r∑i=1nksign(Ωk)(M¯ki−Ωk)nDE,
 MathType@MTEF@5@5@+=feaafiart1ev1aaatCvAUfKttLearuWrP9MDH5MBPbIqV92AaeXatLxBI9gBaebbnrfifHhDYfgasaacH8akY=wiFfYdH8Gipec8Eeeu0xXdbba9frFj0=OqFfea0dXdd9vqai=hGuQ8kuc9pgc9s8qqaq=dirpe0xb9q8qiLsFr0=vr0=vr0dc8meaabaqaciaacaGaaeqabaqabeGadaaakeaacuWGIbGygaqcamaaBaaaleaacqWGebarcqWGfbqraeqaaOGaeyypa0ZaaSaaaeaadaaeWbqaamaaqahabaGaem4CamNaemyAaKMaem4zaCMaemOBa42aaeWaaeaacqqHPoWvdaWgaaWcbaGaem4AaSgabeaaaOGaayjkaiaawMcaaaWcbaGaemyAaKMaeyypa0JaeGymaedabaGaemOBa42aaSbaaWqaaiabdUgaRbqabaaaniabggHiLdGcdaqadaqaaiqbd2eanzaaraWaaSbaaSqaaiabdUgaRjabdMgaPbqabaGccqGHsislcqqHPoWvdaWgaaWcbaGaem4AaSgabeaaaOGaayjkaiaawMcaaaWcbaGaem4AaSMaeyypa0JaeGymaedabaGaemOCaihaniabggHiLdaakeaacqWGUbGBdaWgaaWcbaGaemiraqKaemyraueabeaaaaGccqGGSaalaaa@59AA@

where M¯ki
 MathType@MTEF@5@5@+=feaafiart1ev1aaatCvAUfKttLearuWrP9MDH5MBPbIqV92AaeXatLxBI9gBaebbnrfifHhDYfgasaacH8akY=wiFfYdH8Gipec8Eeeu0xXdbba9frFj0=OqFfea0dXdd9vqai=hGuQ8kuc9pgc9s8qqaq=dirpe0xb9q8qiLsFr0=vr0=vr0dc8meaabaqaciaacaGaaeqabaqabeGadaaakeaacuWGnbqtgaqeamaaBaaaleaacqWGRbWAcqWGPbqAaeqaaaaa@30CD@ are the average normalized log-ratios taken over all arrays, and where *n*_*DE *_is the total number of DE-spots on the array. The selected bias is similarly estimated as the reflected bias albeit only genes classified as DE are used to estimate the bias. For some problems, the selected bias may be a more relevant measure than the reflected bias. In order to determine if the average normalized log-ratios have a strong linear relationship to the true log-ratios, it is necessary to study the bias of the DE-genes individually. The overall standard deviation of the DE-genes can be estimated by

s^DE=∑k=1r∑i=1nk(M¯ki−M¯¯k)2nDE−r,
 MathType@MTEF@5@5@+=feaafiart1ev1aaatCvAUfKttLearuWrP9MDH5MBPbIqV92AaeXatLxBI9gBaebbnrfifHhDYfgasaacH8akY=wiFfYdH8Gipec8Eeeu0xXdbba9frFj0=OqFfea0dXdd9vqai=hGuQ8kuc9pgc9s8qqaq=dirpe0xb9q8qiLsFr0=vr0=vr0dc8meaabaqaciaacaGaaeqabaqabeGadaaakeaacuWGZbWCgaqcamaaBaaaleaacqWGebarcqWGfbqraeqaaOGaeyypa0ZaaOaaaeaadaWcaaqaamaaqahabaWaaabCaeaadaqadaqaaiqbd2eanzaaraWaaSbaaSqaaiabdUgaRjabdMgaPbqabaGccqGHsislcuWGnbqtgaqegaqeamaaBaaaleaacqWGRbWAaeqaaaGccaGLOaGaayzkaaWaaWbaaSqabeaacqaIYaGmaaaabaGaemyAaKMaeyypa0JaeGymaedabaGaemOBa42aaSbaaWqaaiabdUgaRbqabaaaniabggHiLdaaleaacqWGRbWAcqGH9aqpcqaIXaqmaeaacqWGYbGCa0GaeyyeIuoaaOqaaiabd6gaUnaaBaaaleaacqWGebarcqWGfbqraeqaaOGaeyOeI0IaemOCaihaaaWcbeaakiabcYcaSaaa@52E7@

where M¯¯k
 MathType@MTEF@5@5@+=feaafiart1ev1aaatCvAUfKttLearuWrP9MDH5MBPbIqV92AaeXatLxBI9gBaebbnrfifHhDYfgasaacH8akY=wiFfYdH8Gipec8Eeeu0xXdbba9frFj0=OqFfea0dXdd9vqai=hGuQ8kuc9pgc9s8qqaq=dirpe0xb9q8qiLsFr0=vr0=vr0dc8meaabaqaciaacaGaaeqabaqabeGadaaakeaacuWGnbqtgaqegaqeamaaBaaaleaacqWGRbWAaeqaaaaa@2F89@ is the average taken over all arrays and replicates.

### Description of the data analyses used in the evaluation study

Eight hybridized spike-in Lucidea arrays were scanned at four settings (laser power/PMT): 70/70, 80/80, 90/90, and 100/100 (in that order), where the numbers were percentages of maximum values. These scans are referred to as the 70, 80, 90, and 100 scans. The pre-processing procedures considered in this work involved seven consecutive steps: image analysis, filtration, background adjustment, merging data from several scans, channel adjustment, censoring, and calculations of test-statistics (Figure 1). The analyses were carried out using ScanArrayExpress 2.1 (PerkinElmer), Bioconductor [7], the Aroma package [8], and the in-house S-Plus library UmeaSAMED. The evaluations were carried out using the in-house S-Plus library EDMA [9].

### Image analysis

The standard way to conduct image analysis is to analyze the two images obtained from one scan together, so that the spots are equally defined for both channels. We propose an alternative method, the *combined image analysis *where the scan's images are analyzed with images from a second scan (commonly the highest scan in the experiment, in our case the 100 scan), so that the spots are equally defined for all four images. This approach is possible since ScanArray Express allows four images to be analyzed simultaneously. All the 252 evaluated analyses used combined image analysis. The median of the spots' pixel values was used to calculate the intensities. For one array, additional image analyses were done in the standard way using both ScanArrayExpress 2.1 and GenePix 5.0 (Axon Instruments Inc); the software generate "flags" indicating whether the spots are properly identified [10,11]. The percentage of so-called not-found spots was used to characterize the different image analyses (Table 1). Combined image analysis using additional images from a higher scan will improve spot finding and thereby improve the quality of the data.

### Filtration

Intensities from *not-found spots *(*i.e*. spots not properly identified by the image analysis software) were treated in three different ways:

I. *Complete filtration: *the intensities were treated as missing values.

II. *Partial filtration*: the intensities were treated as missing values during normalization, but prior to calculating test-statistics the spot's log-ratios were set to zero. In the special case when all arrays generated not-found spots, the gene was removed from the experiment.

III. *No filtration: *the intensities were treated as intensities of found spot.

Complete filtration is commonly used while partial filtration is a novel method. The idea behind partial filtration is that spots called "not found" commonly arise from genes that are not expressed in either channel, and therefore can be regarded as NDE-genes.

### Background adjustment

The analyses either did not apply any background adjustment, or applied the standard background adjustment removing the local background intensities from the observed intensities. Background adjustment divided the spots into three groups: *A-spots*, where both the reference and the treated background adjusted intensities (ba-intensities) were positive, *B-spots *where either the reference or the treated ba-intensity was negative, and *C-spots *where both the ba-intensities were negative.

### Merging data from several scans

Scans generated 16-bit images and, since the median was used to calculate the spot intensities, all intensities were integers between 0 and 2^16 ^-1. Henceforth, intensities equal to the maximum value will be called *saturated*. One common approach to deal with saturation is to adjust the scanner settings such that only a small fraction of the intensities will be saturated. Two alternative approaches, *restricted linear scaling *(RLS) and *the constrained model *(CM) [12], combine intensities from two or more scans in order to expand the linear range of the experiment. RLS is a slight modification of the algorithm suggested by Dudley *etal*. [13]. Seven scanning procedures were considered:

I. Using data from the 70 scan.

II. Using data from the 80 scan.

III. Using data from the 90 scan.

IV. Using data from the 100 scan.

V. RLS using combined data from the 70, 80, and 90 scans (RLS 90).

VI. RLS using combined data from the 80, 90, and 100 scans (RLS 100).

VII. CM using data from all four scans with the 70 data as baseline.

The 80 scan can be thought of as a standard scan since it was the highest scan where only a small fraction (< 0.2%) of the intensities were saturated. From a practical point of view, the CM and RLS procedures demand more scanning and image analyses. In addition, databases created for microarray data storage do not commonly support data from several scans, *e.g*., BASE [14]. The storage problem can usually be solved by creating additional software [15].

### Channel adjustment

The print-tip lowess normalization [16] was used to remove the systematic differences between channels. For the Lucidea experiment, only data from NDE-genes were used to fit the lowess curves. However, data from all A-spots were adjusted. Clearly, this approach is an idealization, since the DE-genes in a real experiment affect the estimated curves. However, if the proportion of DE-genes is small, and if the true log-ratios are symmetrically distributed around zero, then this effect is small. For two analyses different proportions of DE-genes were used to fit the lowess curves. For these analyses, inclusion of a small number of DE-genes had marginal effect on the analyses' sensitivities and biases (Table 2). Intensities from B-spots were treated as missing values, while intensities from C-spots were treated in two different ways:

I. *Removed*: the C-spots' intensities were treated as missing values.

II. *Included: *the C-spots' log-ratios were set to zero prior to calculating the test-statistics.

The rationale for including C-spots is again that these spots commonly arise from genes that are not expressed in either channel and therefore have mRNA log-ratios equal to zero.

### Censoring of low intensities

A-spot intensities were censored such that all intensities below a user-defined censoring value *λ *were increased to this value. In this work the censoring values 1, 8, 64, and 512 were used. In practice, using *minimal censoring *(*i.e. λ *= 1) is equivalent to applying no censoring at all. The idea behind censoring is to moderate the variance of the weakly expressed genes. It still remains to determine how to select an optimal censoring value. In this paper, background adjustment is a spot level procedure whereas censoring is an adjustment applied globally to an array.

### Test-statistics

The statistics generated by the B-test were used as test-statistics.

### Empirical results of the evaluation study

The data generated by the eight hybridized Lucidea arrays were normalized in 252 ways as described in Table 3. The notation S.P.*λ *refers to a normalization that used scanning procedure S and *procedure *P (I-IX in Table 3) with censoring value *λ*. The censoring value (among 1, 8, 64, and 512) generating the highest sensitivity for a group of analyses using scanning approach S and procedure P, will be referred to as the groups *optimal censoring value*.

**Figure 3:**
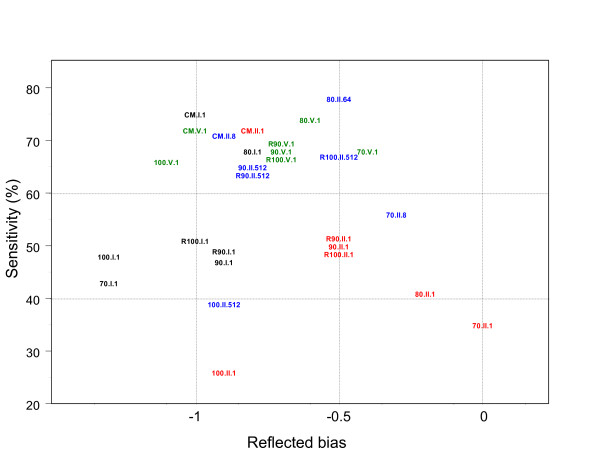
**Reflected bias and sensitivity for selected normalizations**. Reflected bias and sensitivity for 24 normalizations using four types of procedures for filtration, background adjustment, and censoring: complete filtration without background adjustment and censoring (black), complete filtration with background adjustment but without censoring (blue), complete filtration with background adjustment and optimal censoring (red), and partial filtration without background adjustment and censoring (green).

**Figure 4:**
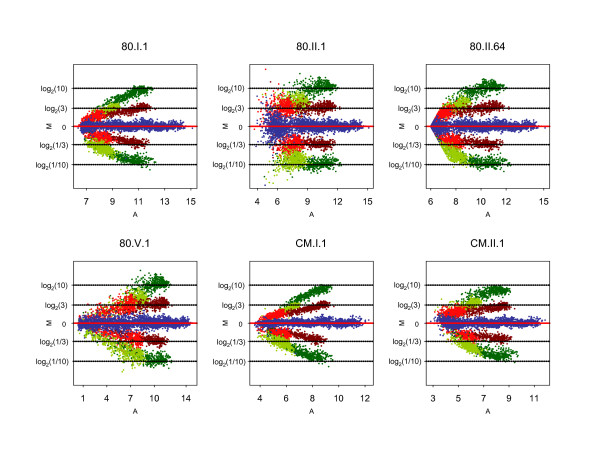
**MA-plots for the selected normalizations**. MA-plots for six normalizations. The coloring corresponds to genes 1–18 in Figure 2: NDE-genes (1–10) are blue, highly-regulated genes (*i.e*. ratio 1/10 or 10) expressed at high concentrations (12, 18) are dark green, highly-regulated genes expressed at low concentrations (11, 17) are light green, moderately-regulated genes (*i.e*. ratio 1/3 or 3) expressed at high concentrations (14, 16) are brown, and moderately-regulated genes expressed at low concentrations (13, 15) are colored red.

**Figure 5:**
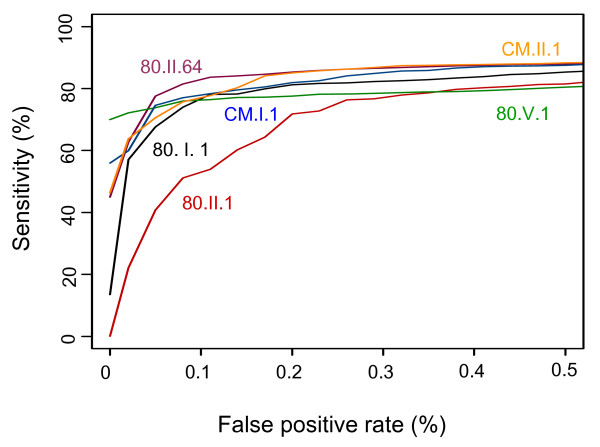
**ROC-curves for selected normalizations**. ROC-curvesfor six normalizations; 80.I.1 (black), 80.II.1 (red), 80.II.64 (darkred), 80.V.1 (green), CM.I.l (blue), and CM.II.l (yellow).

**Figure 6:**
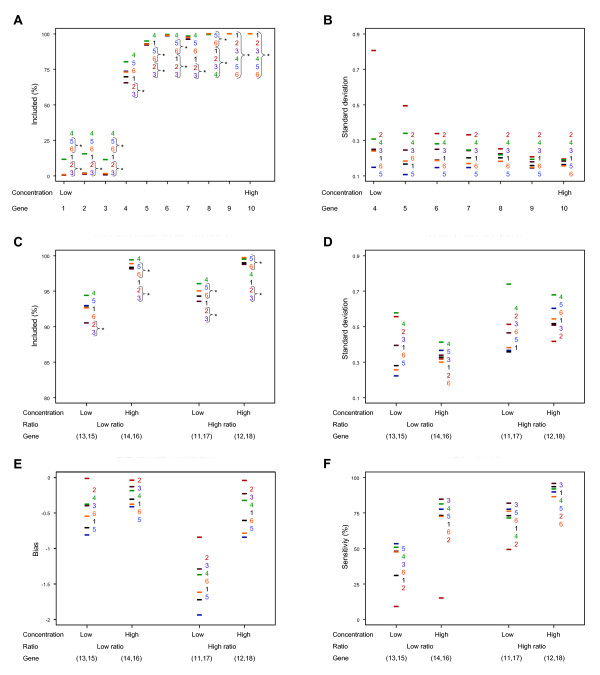
**Description of individual genes for selectednormalizations**. Six measures are presented: the percentage of NDE-genes included^1 ^in the statistical analysis (6A), the overall standard deviation of the NDE-genes (6B), the percentage of DE-genes included^1 ^in the statistical analysis (6C), the overall standard deviation of the DE-genes (6D), the reflected bias of the DE-genes (6E), and the sensitivity of the DE-genes (6F). Details of the genes (1–18) are found in Figure 2. In 6C-6F, genes with the same magnitude of regulation are presented together. The analyses are marked with different numbers: 80.I.1 (1), 80.II.1 (2), 80.II.64 (3), 80.V.1 (4), CM.I.l (5) and CM.II.l (6). A star indicates that values are equal. 1) A gene was *included *in the statistical analysis if at least one of the arrays was able to generate an observation of the desired log-ratio. In addition, analyses that used partial filtration did not include genes for which all arrays generated so-called not-found spots.

For all normalizations the properties of the analyzed data were summarized by observing the sensitivity at the 0.05%, 0.1%, and 0.5% FPRs (Table 4, 5, 6), the overall reflected bias (Table 7), and the overall standard deviation (Table 8). Figure 3 shows the bias and sensitivity for an interesting subclass of analyses. Six normalizations were selected as particularly interesting and were investigated in some detail (Table 9, Figure 4, 5, 6).

Normalizations without background adjustment did not benefit from censoring, therefore only results observed at the minimal censoring are presented in this paper. The number of C-spots was low when either partial or complete filtration was used (data not shown), and consequently including C-spots (III and VI) gave similar results to excluding C-spots (II and V), so only results from the later analyses are presented in this paper. The partial filtration with background adjustment (V) performed considerable better (higher sensitivity and lower bias) than the partial filtration without background adjustment (IV) (Table 4, 5, 6, 7). Normalizations without filtration (VII, VIII, and IX) and minimal censoring had very low sensitivities. In this case, optimal censoring gave considerable higher sensitivities, but these were still lower than the sensitivities obtained when procedure V was used (Table 4, 5, 6, 7).

Henceforth, we concentrate on what are arguably the four most interesting procedures: complete filtration without background adjustment (I), complete filtration with background adjustment and minimal censoring (II.1), complete filtration with background adjustment and optimal censoring (II.op), and partial filtration with background adjustment (V). These procedures were combined with the seven scanning procedures to give a subclass of 24 normalizations. The properties of these normalizations are discussed below.

### Overall bias and standard deviation

For all except one of the analyses (with 70.II.1 as the exception), the reflected bias was negative and the magnitude of the regulation was underestimated (Table 7). Background adjustment had a positive effect on the bias, while censoring and the use of partial filtration resulted in high bias (Table 7, Figure 3). The use of the 70 and 80-scan procedures resulted in relatively small bias, while the CM-procedure gave high bias (Table 7, Figure 3). The standard deviation was generally high among normalizations using partial filtration, and low among methods using complete filtration without background adjustment or the CM-procedure (Table 8).

### Sensitivity at the 0.05% false positive rate

Normalizations that used complete filtration and minimal censoring (I and II. 1) had generally low sensitivities (26–51%) (the exceptions were analyses 80.I.1, CM.I.l, and CM.II.l), while analyses that used partial filtration had relatively high sensitivities (66–74%) (Table 4, Figure 3). Normalizations that used the CM-procedure or the 80-scan procedure (except 80.II.1) had high sensitivities (68–78%) (Table 4, Figure 3).

### Sensitivity at the 0.1 and 0.5% false positive rates

The results at the 0.1% FPR were similar to those obtained at the 0.05% FPR, although the variability between the analyses was smaller (Table 5). The variability at the 0.5% FPR was even smaller, and the analyses' sensitivities varied between 65 and 88% (Table 6). Normalizations that used the 70 or 100-scan procedure had with one exception (70.II.8) the smallest sensitivities, while analyses that used the CM-procedure had among the highest sensitivities (Table 6).

### A detailed comparison between six selected normalizations

Four of the best performing normalizations, 80.II.64, 80.V.I, CM.I.l, and CM.II.l, together with 80.I.1 and 80.II.1 (arguably standard normalizations), were selected for further comparison. The MA-plots (average normalized log-intensities versus the average normalized log-ratios) for the six analyses are shown in Figure 4. Note the characteristic rocket shape formed by the NDE-intensities for the analysis 80.II.1; this analysis had low bias and low sensitivity (Table 9, Figure 5). The other analyses avoided the typical rocket shape and thereby achieved higher sensitivity but also higher bias (Table 9, Figure 4). The trade-off between an analysis' ability to identify DE-genes and its ability to obtain low bias has previously been discussed, *e.g*. [5].

A large proportion of the genes expressed at very low concentrations were removed prior to the test, independent of which normalization was used (Figure 6A). For analysis 80.II.1, the standard deviation of the NDE-genes decreased with the mRNA-concentration, indicating that the majority of the extreme NDE-log-ratios were caused by genes expressed at low concentrations (Figure 6B).

For analysis 80.V.1 the selected bias (estimated by data from correctly classified DE-genes) was considerable lower than the reflected bias (Table 9). It follows that analysis 80.V. 1 divides the DE-genes into two categories; the correctly classified genes with relatively low bias and the falsely classified genes with high bias. Genes with several so-called not found spots are likely to fall in the second category. Spot finding was positively correlated with the mRNA-concentration (Figure 6A,C), and DE-genes with low mRNA-concentrations had higher bias, higher standard deviation, and lower sensitivity than genes with high mRNA-concentrations (Figure 4, 6D,E,F).

For most of the other normalizations, DE-genes expressed at low concentrations had considerable higher bias and lower sensitivity than genes expressed at high concentrations (Figure 4, 6E,F). Highly-regulated genes (DE-genes that were either tenfold up- or down-regulated) expressed at low concentrations (*i.e*. 11 and 17 in Figure 2) had the highest bias (Figure 6E). These genes were expressed at lower concentrations than any of the other DE-genes. Interestingly, the highly up-regulated genes 12 and 18 had equal or higher bias than the moderately-regulated genes 14 and 16 even though they were expressed at higher concentrations. This suggests that the magnitude of the regulation affects the bias so that highly-regulated genes generally have higher bias than moderately-regulated genes.

## Discussion

In some important aspects our experiment differs from an ordinary microarray experiment. Most importantly, the Lucidea data were not influenced by any biological variation. It is unclear how adding biological variation influences the relative ranking of the evaluated analyses. Furthermore, the evaluation is based on eight arrays. It is possible that the number of arrays in an experiment affects the relative ranking of the analyses. Non-expressed genes that are switched on and expressed genes that are switched off can be very interesting. Genes of this type are not present on the Lucidea array. From an experimental point of view we have the complication that each array was scanned at four settings (laser power/PMT): 70/70, 80/80, 90/90, and 100/100 (in that order), and it is possible that data from the higher settings lost some information due to photobleaching. The sensitivities, biases, and standard deviations presented in this paper are all point estimators, the uncertainties of these estimates are not considered in this paper. Despite these limitations, the Lucidea experiment gives valuable information about the relatively performances of the evaluated analysis methods.

In microarray analyses, one of the most important and difficult problems is to select a cut-off value in order to control the false discovery rate. This problem is not considered in this paper.

The 252 evaluated analyses represent only a small fraction of all available pre-processing procedures. The background adjustment, the print-tip lowess normalization, and the B-test used in the evaluation are all widely used methods, but not necessarily the best methods available. For example, it is possible that better results can be obtained using more advanced background adjustment procedures [17]. Furthermore, the inclusion of C-spots, partial filtration, and censoring generate log-ratios that are affected by censored intensities. Although the B-test is a robust test [2], it was not designed to handle censored observations. All analyses used the same type of image analysis. It is possible that there exist image analysis methods with significantly better spot finding properties than ScanArrayExpress and that the use of such methods could change the relative ranking of the evaluated normalization procedures.

Both partial filtration and the inclusion of C-spots are built on the idea that not-found spots and C-spots are most likely observations from non-expressed genes. However, occasionally these spots arise due to experimental failures. The probability that not-found spots and C-spots arise from non-expressed genes might be determined by considering observations from all arrays simultaneously. Analyses using this information are likely to perform better than the methods suggested in this paper.

Considerable space was devoted to censoring. Even though there is no method for determining the optimal censoring values. However, censoring increased the sensitivities of most analyses using background adjustment, sometimes dramatically. We find these results promising and think that they can serve as an inspiration for further research.

## Conclusion

The use of spike-in experiments is a powerful approach for evaluating microarray preprocessing procedures. The sensitivities at low false positive rates and the reflected bias are useful measures for characterizing analyzed microarray data.

In general, there was a trade-off between the ability of the analyses to identify DE-genes and their ability to provide unbiased estimators of the desired ratios. No single analysis achieved both low bias and high sensitivity. The magnitude of the regulation of the DE-genes was underestimated by almost all analyses, often less than 50% (*i.e*. reflected bias < -1) of the true regulation were observed. Moreover, the bias depended on the underlying mRNA-concentrations; DE-genes with low concentration generally had higher bias than genes expressed at high concentration.

When very low false positive rates were considered (*e.g*. 0.05%); many of the analyses had relatively low sensitivities. However, analyses that used either the CM-procedure or partial filtration had with few exceptions high or very high sensitivities. If bias is not a major problem; we strongly recommend the use of either the CM-procedure or partial filtration, which gives considerable higher sensitivities than some commonly used analysis methods.

## Methods

### The Lucidea arrays

The arrays were in-house produced cDNA-arrays [18] consisting of 20 clones from the Lucidea Universal ScoreCard (Figure 2). The clones were dissolved in 50% DMSO and printed on UltraGAPS slides (Corning Life Science) using a Microgrid II arrayer (Biorobotics). Each clone was printed 480 times in 48 identically designed sub-grids. Besides the Lucidea genes several other genes were printed on the array, but data from these genes was excluded prior to normalization.

### Labeling, hybridization, and scanning

Eight Lucidea arrays were hybridized. In short, first strand cDNA-synthesis was performed using Superscript II (Invitrogen) incorporating aminoallyl-dUTP (Amersham Biosciences). Five *μ*l of the Lucidea Universal ScoreCard reference and test spike mix RNA, together with 25 *μ*g of total RNA from murine cell line J774.1, were used in the respective reactions. The fluorophores Cy3 and Cy5 (Amersham Biosciences) were coupled to the aminoallyl group in the test and reference reactions respectively. The labeled cDNA was purified, and cDNA from test and reference reactions were mixed and dissolved in DIG Easy Hyb (Roche) supplemented with tRNA (Sigma) and fish sperm DNA (Sigma). The arrays were hybridized overnight at 37°C. Washing was performed in a Genetac hybridization station (Genomic Solutions) at 50°C in 0.1 × SSC, 0.1% SDS, followed by 0.1 × SSC at 20°C. Each array was scanned using a ScanArray 4000XL (PerkinElmer) at four different settings.

### Image analysis

The Images were analyzed by ScanArrayExpress, using adaptive circle with nominal diameter 150 *μ*m. The median was used to calculate both the foreground and background intensities. One array was also analyzed by Genepix using the circular feature with nominal diameter 150 *μ*m.

## Authors' contributions

PR and LNO designed the Lucidea array. LN and LNO produced the arrays. LN carried out the laboratory work. BH performed the image analyses. HA invented the combined image analysis method and PR conceived the other novel analysis methods presented in this work. ML and PR created EDMA and carried out the evaluation study. All authors helped to draft the manuscript.

## Figures and Tables

**Table 1:** Combined and standard image analysis. For one of the scanned Lucidea arrays, image analyses were performed in three different ways; standard analysis using GenePix, standard analysis using ScanArrayExpress, and combined analysis using ScanArrayExpress. The standard method analyzed images from one scan so that the spots were equally defined for both channels, while the combined method analyzed images from two scans so that the spots were equally defined for all four images. Here "70+100" means that images from the 70 scan were analyzed together with images from the 100 scan. The table shows the percentage of spots that the image analyses failed to identify.

	ScanArrayExpress							GenePix
	
	Standard				Combined			Standard
Scan	**70**	**80**	**90**	**100**	**70+100**	**80+100**	**90+100**	**80**
Not found (%)	52	49	44	41	41	41	37	46

**Table 2:** Print-tip lowess normalization. In two of the analyses (80.I.1 and 80.II.1, see Table 3), the percentage of DE-genes allowed to influence the lowess curve estimates was kept at five different levels; 0, 1,6, 11, and 40%. The latter was obtained when all DE-genes were included. For each analysis, the sensitivities at the 0.05, 0.1, and 0.5% FPRs were observed together with the reflected bias.

DE-genes (%)	80.I.1					80.II.1				
	
	**0**	**1**	**6**	**11**	**40**	**0**	**1**	**6**	**11**	**40**
Sensitivity (%) at 0.05% FPR	68	68	68	68	47	41	41	41	36	31
Sensitivity (%) at 0.1% FPR	78	78	78	78	60	54	54	51	48	37
Sensitivity (%) at 0.5% FPR	85	86	86	86	70	81	81	81	82	69
Reflected bias	-0.8	-0.8	-0.8	-0.8	-0.9	-0.2	-0.2	-0.2	-0.2	-0.4

**Table 3:** Description of the analyses used in the evaluation study. The nine procedures for filtration, background adjustment, and channel adjustment that were evaluated. These procedures were combined with seven scanning procedures (70, 80, 90, 100, RLS 90, RLS 100, and CM) and four censoring values (1, 8, 64, and 512). Combined image analysis using ScanArrayExpress, print-tip lowess, and the B-test were used by all analyses.

	Procedure								
	
	**I**	**II**	**III**	**IV**	**V**	**VI**	**VII**	**VIII**	**IX**
Filtration	Complete filtration			Partial filtration			No filtration		

Background adjustment	No	Yes	Yes	No	Yes	Yes	No	Yes	Yes
C-spot adjustment	Excl.	Excl.	Incl.	Excl.	Excl.	Incl.	Excl.	Excl.	Incl.

**Table 4:** Sensitivity at the 0.05% false positive rate. The estimated sensitivity (%) obtained when the FPR was fixed at 0.05%. If approximately 1% of the genes are DE, then an observed sensitivity in the range of 40–80% converts to an observed false discovery rate of between 5 and 11%.

Scanning procedure	Censuring value	Procedure						
		
		Complete filtration		Partial filtration		No filtration		
		
		No ba	Ba	No ba	Ba	No ba	Ba	Ba+C
		
		**I**	**II**	**IV**	**V**	**VII**	**VIII**	**IX**
70 scan	1	43	35	37	68	29	27	48
	8		56		57		53	61
	64		38		27		36	30
	512		2		0		1	0

80 scan	1	68	41	65	74	58	5	24
	8		41		72		20	43
	64		78		65		70	68
	512		34		28		28	27

90 scan	1	47	50	55	69	37	2	21
	8		50		66		2	21
	64		38		65		24	42
	512		65		57		43	42

100 scan	1	48	26	55	66	27	0	2
	8		26		62		0	2
	64		26		63		0	2
	512		39		63		46	51

RLS 90	1	49	51	65	69	57	5	20
	8		52		64		8	22
	64		35		64		24	42
	512		64		53		54	55

RLS 100	1	51	49	60	67	44	5	12
	8		49		63		5	12
	64		49		64		6	13
	512		67		63		52	67

CM	1	75	71	63	72	45	45	52
	8		72		68		73	73
	64		41		33		29	29
	512		3		1		1	1

**Table 5:** Sensitivity at the 0.1 % false positive rate. The estimated sensitivity (%) obtained when the FPR was fixed at 0.1%.

Scanning procedure	Censuring value	Procedure						
		
		Complete filtration		Partial filtration		No filtration		
		
		No ba	Ba	No ba	Ba	No ba	Ba	Ba+C
		
		**I**	**II**	**IV**	**V**	**VII**	**VIII**	**IX**
70 scan	1	50	45	42	71	36	40	61
	8		60		63		69	65
	64		43		33		40	34
	512		2		1		1	1

80 scan	1	78	54	67	76	64	10	29
	8		55		73		22	62
	64		84		68		75	72
	512		38		30		32	30

90 scan	1	60	57	56	73	48	7	31
	8		57		69		6	32
	64		53		69		28	52
	512		70		58		48	47

100 scan	1	58	37	57	70	29	1	7
	8		37		67		1	6
	64		37		67		2	8
	512		46		66		52	58

RLS 90	1	70	59	70	71	59	14	29
	8		59		67		14	31
	64		56		66		28	52
	512		67		63		59	60

RLS 100	1	68	59	63	72	53	14	16
	8		59		67		14	16
	64		59		68		14	16
	512		71		68		66	71

CM	1	78	78	68	75	53	51	67
	8		78		71		75	74
	64		46		38		33	33
	512		4		2		2	2

**Table 6:** Sensitivity at the 0.5% false positive rate. The estimated sensitivity (%) obtained when the FPR was fixed at 0.5%.

Scanning procedure	Censuring value	Procedure						
		
		Complete filtration		Partial filtration		No filtration		
		
		No ba	Ba	No ba	Ba	No ba	Ba	Ba+C
		
		**I**	**II**	**IV**	**V**	**VII**	**VIII**	**IX**
70 scan	1	68	73	59	76	53	65	76
	8		81		72		79	74
	64		53		44		51	44
	512		12		8		10	8

80 scan	1	85	81	75	80	77	27	68
	8		82		79		41	72
	64		88		74		82	79
	512		46		38		40	39

90 scan	1	79	78	67	79	57	21	53
	8		79		76		21	54
	64		79		76		53	69
	512		80		67		56	55

100 scan	1	74	65	67	76	38	9	29
	8		65		74		9	29
	64		65		74		10	28
	512		67		73		65	68

RLS 90	1	85	79	78	80	76	23	53
	8		79		77		22	54
	64		80		77		50	69
	512		82		71		72	71

RLS 100	1	82	82	75	80	73	23	47
	8		82		77		23	46
	64		82		77		23	45
	512		81		77		76	77

CM	1	88	88	77	81	73	74	76
	8		88		79		84	84
	64		57		47		42	42
	512		14		9		9	9

**Table 7:** Reflected bias. The estimated reflected bias of the DE-genes as defined in Relation 2. A reflected bias equal to -1 implies that only 50% of the true regulation was observed.

Scanning procedure	Censuring value	Procedure						
		
		Complete filtration		Partial filtration		No filtration		
		
		No ba	Ba	No ba	Ba	No ba	Ba	Ba+C
		
		**I**	**II**	**IV**	**V**	**VII**	**VIII**	**IX**
70 scan	1	-1.3	0.0	-1.4	-0.4	-1.5	-0.2	-0.4
	8		-0.3		-0.7		-0.4	-0.6
	64		-1.2		-1.4		-1.3	-1.4
	512		-2.2		-2.2		-2.2	-2.2

80 scan	1	-0.8	-0.2	-1.0	-0.6	-1.0	-0.4	-0.4
	8		-0.3		-0.6		-0.4	-0.4
	64		-0.5		-0.8		-0.7	-0.7
	512		-1.4		-1.5		-1.5	-1.5

90 scan	1	-0.9	-0.5	-1.0	-0.7	-1.0	-0.6	-0.6
	8		-0.5		-0.7		-0.6	-0.6
	64		-0.5		-0.8		-0.6	-0.6
	512		-0.8		-1.0		-0.9	-1.0

100 scan	1	-1.3	-0.9	-1.4	-1.1	-1.4	-1.0	-1.0
	8		-0.9		-1.1		-1.0	-1.0
	64		-0.9		-1.1		-1.0	-1.0
	512		-0.9		-1.1		-1.1	-1.1

RLS 90	1	-0.9	-0.5	-1.0	-0.7	-1.0	-0.6	-0.6
	8		-0.5		-0.7		-0.6	-0.6
	64		-0.5		-0.7		-0.6	-0.6
	512		-0.8		-1.0		-0.9	-0.9

RLS 100	1	-1.0	-0.5	-1.1	-0.7	-1.1	-0.6	-0.6
	8		-0.5		-0.7		-0.6	-0.6
	64		-0.5		-0.7		-0.6	-0.6
	512		-0.5		-0.8		-0.7	-0.7

CM	1	-1.0	-0.8	-1.1	-1.0	-1.1	-0.7	-0.7
	8		-0.9		-1.0		-0.8	-0.8
	64		-1.4		-1.5		-1.5	-1.5
	512		-2.2		-2.2		-2.2	-2.2

**Table 8:** Standard deviation of the DE-genes. The estimated overall standard deviation of the DE-genes as defined in Relation 3.

Scanning procedure	Censuring value	Procedure						
		
		Complete filtration		Partial filtration		No filtration		
		
		No ba	Ba	No ba	Ba	No ba	Ba	Ba+C
		
		**I**	**II**	**IV**	**V**	**VII**	**VIII**	**IX**
70 scan	1	0.40	0.55	0.46	0.71	0.46	0.61	0.73
	8		0.50		0.73		0.59	0.69
	64		0.52		0.57		0.54	0.56
	512		0.30		0.28		0.29	0.28

80 scan	1	0.38	0.46	0.51	0.61	0.49	0.59	0.60
	8		0.44		0.66		0.56	0.58
	64		0.43		0.62		0.56	0.58
	512		0.52		0.54		0.53	0.53

90 scan	1	0.41	0.53	0.49	0.59	0.46	0.59	0.60
	8		0.52		0.62		0.57	0.58
	64		0.46		0.60		0.52	0.53
	512		0.49		0.59		0.56	0.57

100 scan	1	0.40	0.53	0.44	0.58	0.39	0.61	0.61
	8		0.53		0.61		0.61	0.61
	64		0.51		0.60		0.58	0.58
	512		0.47		0.58		0.53	0.53

RLS 90	1	0.42	0.52	0.50	0.59	0.47	0.59	0.60
	8		0.51		0.62		0.57	0.58
	64		0.46		0.60		0.52	0.53
	512		0.50		0.59		0.57	0.58

RLS 100	1	0.41	0.49	0.48	0.58	0.46	0.60	0.60
	8		0.49		0.61		0.59	0.59
	64		0.48		0.60		0.56	0.57
	512		0.44		0.59		0.52	0.53

CM	1	0.42	0.39	0.48	0.48	0.48	0.47	0.48
	8		0.39		0.51		0.51	0.51
	64		0.47		0.49		0.51	0.51
	512		0.26		0.26		0.25	0.25

**Table 9:** Estimated measures for six selected analyses. The table shows sensitivity (%), reflected bias, and selected bias for six analyses.

	Analysis					
	
	**80.I.1**	**80.II.1**	**80. II. 64**	**80.V.1**	**CM. I.1**	**CM. II.1**
FPR (%)	Sensitivity (%)					

0.05	68	41	78	74	75	71
0.1	78	54	84	76	78	78
0.5	85	81	88	80	88	88

	Reflected bias					
	
	-0,8	-0,2	-0,5	-0,6	-1,0	-0,8
	
	Selected bias					

0.05	-0,7	-0,1	-0,4	-0,3	-0,9	-0,7
0.1	-0,7	-0,1	-0,4	-0,3	-0,9	-0,7
0.5	-0,8	-0,2	-0,4	-0,4	-0,9	-0,8
